# Architecture of Parallel Adaptation in Ten Lacustrine Threespine Stickleback Populations from the White Sea Area

**DOI:** 10.1093/gbe/evz175

**Published:** 2019-08-12

**Authors:** Nadezhda V Terekhanova, Anna E Barmintseva, Alexey S Kondrashov, Georgii A Bazykin, Nikolai S Mugue

**Affiliations:** 1 Skolkovo Institute of Science and Technology, Skolkovo, Russia; 2 Sector for Molecular Evolution, Institute for Information Transmission Problems of the RAS (Kharkevich Institute), Moscow, Russia; 3 Laboratory of Molecular Genetics, Russian Federal Research Institute of Fisheries and Oceanography, Moscow, Russia; 4 Department of Ecology and Evolutionary Biology, University of Michigan, Ann Arbor, Michigan; 5 M. V. Lomonosov Moscow State University, Moscow, Russia; 6 N. K. Koltzov Institute of Developmental Biology RAS, Moscow, Russia

**Keywords:** molecular evolution, adaptation, divergence islands

## Abstract

Adaptation of threespine stickleback to freshwater involves parallel recruitment of freshwater alleles in clusters of closely linked sites, or divergence islands (DIs). However, it remains unclear to what extent the DIs and the alleles that constitute them coincide between populations that underwent adaptation to freshwater independently. We examine threespine sticklebacks from ten freshwater lakes that emerged 500–1500 years ago in the White Sea basin, with the emphasis on repeatability of genomic patterns of adaptation among the lake populations and the role of local recombination rate in the distribution and structure of DIs. The 65 detected DIs are clustered in the genome, forming 12 aggregations, and this clustering cannot be explained by the variation of the recombination rate. Only 21 of the DIs are present in all the freshwater populations, likely being indispensable for successful colonization of freshwater environment by the ancestral marine population. Within most DIs, the same set of single nucleotide polymorphisms (SNPs) distinguish marine and freshwater haplotypes in all the lake populations; however, in some DIs, freshwater alleles differ between populations, suggesting that they could have been established by recruitment of different haplotypes in different populations.

## Introduction

The wide range of threespine stickleback *Gasterosteus aculeatus* encompasses both seas and freshwater bodies in the Northern Hemisphere (Bell and Foster 1994). Its marine populations can rapidly evolve adaptations to freshwater lakes and streams (Terekhanova et al. 2014; Lescak et al. 2015). A newly formed freshwater lake may soon become colonized by individuals of marine ancestry that give rise to the freshwater population (Jones et al. 2012; Roesti et al. 2015; Marques et al. 2016). This process often occurs independently in different lakes (Colosimo et al. 2005). If the connection between the lake and the sea is not severed, gene flow between the ancestral marine and the derived freshwater population may persist (Roesti et al. 2015; Pedersen et al. 2017).

Marine and freshwater environments of threespine stickleback are drastically different, and different salinity, parasites, and predators exert divergent selective pressures on the corresponding populations. Nevertheless, adaptation of a newly established resident population to the lacustrine environment often proceeds very fast, over the course of several decades (Terekhanova et al. 2014; Lescak et al. 2015; Marques et al. 2018), with some changes becoming detectable even sooner (Barrett et al. 2008). Clearly, such rapid complex adaptation cannot depend on de novo mutations (Orr 1998) and must rely primarily on standing genetic variation (Schluter and Conte 2009; Karasov et al. 2010; Matuszewski et al. 2015). Indeed, marine populations of threespine stickleback harbor, at low frequencies, alleles that confer adaptation to freshwater (Schluter and Conte 2009), presumably due to gene flow from the coastal freshwater populations (Bassham et al. 2018). Although such alleles must be deleterious under the unsuitable marine environment, the resulting selection is not strong enough to eliminate them immediately (Bassham et al. 2018). As a result, the sets of alleles that distinguish derived freshwater populations from the ancestral marine population (marker single nucleotide polymorphisms, “marker SNPs”; Terekhanova et al. 2014) are often identical by descent between different lakes (Hohenlohe et al. 2010; DeFaveri et al. 2011; Jones et al. 2012; Terekhanova et al. 2014).

Overall, marine and freshwater genotypes are very similar to each other; however, there are a number of genomic regions where their divergence is considerably higher than the genome average. If only such regions, known as divergence islands (DIs) (Turner et al. 2005; Feder and Nosil 2010), are taken into account, threespine stickleback populations usually become subdivided into marine and freshwater clades (Colosimo et al. 2005; Nelson and Cresko 2018). DIs can be identified by their enrichment with the marker SNPs that have substantially different allele frequencies in the marine and freshwater populations. DIs have been observed in a number of species that recently evolved adaptations to new environments (Ellegren et al. 2012; Jones et al. 2012; Nadeau et al. 2012; Via 2012; Renaut et al. 2013; Riesch et al. 2017).

DIs are often located in genomic regions with reduced recombination (Noor and Bennett 2009; Renaut et al. 2013; Roesti et al. 2013; Feulner et al. 2015; Marques et al. 2016; Samuk et al. 2017) and are characterized by increased linkage disequilibrium (Hohenlohe et al. 2012; Larson et al. 2017), which could be partially due to recent selective sweeps or background selection (Ellegren et al. 2012; Feulner et al. 2015). Local selective sweeps initially cause strong population differentiation in the genomic region around the target of positive selection (Smith and Haigh 1974; Barton 2000). After the allele replacement is over, the length of the affected region decreases with time due to recombination, as long as some migration between the populations persists. Of course, if a gene involved in local adaptation is situated within an inversion, the whole inversion may become a DI (Kirkpatrick 2010; Sodeland et al. 2016). Still, most DIs in sticklebacks are not associated with inversions (Peichel and Marques 2017). A DI can emerge around a single locally adapted locus; however, multiple tightly linked targets of positive selection within a DI are also possible, and it may be difficult to distinguish these two alternatives. It was shown that the distribution of quantitative trait loci across the stickleback genome is significantly nonuniform (Peichel and Marques 2017).

The distribution of positions with high difference in allele frequencies between marine and freshwater populations, marker SNPs, within a DI is also usually nonuniform (Terekhanova et al. 2014), and, as long as some recombination takes place, their density should be higher close to the target(s) of divergent selection. DIs can be ancient (Ma et al. 2018; Nelson and Cresko 2018); the DIs found in threespine stickleback have originated, on average, ∼6 Ma and were shaped by the recurrent action of divergent selection since then (Nelson and Cresko 2018). Over the course of their long history, these DIs have accumulated many marker SNPs that distinguish the marine and the freshwater haplotypes and, in some cases, inversions which suppress recombination between them. When an inversion is present, its boundaries may coincide with the boundaries of the corresponding DI, in which case the density of marker SNPs may be uniform across the whole DI (Jones et al. 2012; McGaugh and Noor 2012; Nadeau et al. 2012; Sodeland et al. 2016).

Data on genotypes of multiple freshwater populations (Hohenlohe et al. 2010; DeFaveri et al. 2011; Jones et al. 2012; Terekhanova et al. 2014) show that a large proportion of the same marker SNPs is present in many, or even all of them. Thus, evolution of adaptations to freshwater proceeds through assembly of “precast bricks” of freshwater-adapted haplotypes which are a part of standing genetic variation in the marine populations (Terekhanova et al. 2014)—a process referred to as “soft sweep” (Orr and Betancourt 2001; Messer and Petrov 2013; Garud et al. 2015). Some data suggest that a diverse set of haplotypes can be involved in adaptation in the same locus at different populations (Roesti et al. 2014; Bassham et al. 2018; Haenel et al. 2019), but this has not been examined systematically.

It has been suggested that the efficiency of divergent selection for a new mutation that is slightly beneficial in one of the two different environments is increased in the vicinity of loci that had already undergone divergent selection for these environments. Theory predicts that this process, termed “divergence hitchhiking” (Via 2009; Feder et al. 2012), can affect the evolution of a DI, leading to their extension (Feder and Nosil 2010; Feder et al. 2012), which is aided by the presence of structural variants that suppress recombination (Flaxman et al. 2013; Yeaman 2013; Yeaman et al. 2016). However, different data analyses provide conflicting estimates of the impact of divergence hitchhiking on the evolution of DIs (Hohenlohe et al. 2012; Renaut et al. 2012; Via 2012; Burri et al. 2015; Feulner et al. 2015).

DIs form clusters (“archipelagos”) within individual chromosomes in different systems (Nadeau et al. 2012; Via 2012; Malinsky et al. 2015; Riesch et al. 2017). This is consistent with divergence hitchhiking (Feder and Nosil 2010; Feder et al. 2012), but can also be explained by the differences in recombination rates between chromosome regions (Berner and Roesti 2017; Samuk et al. 2017; Haenel et al. 2018). Data capable of distinguishing these two mechanisms for establishment of DI archipelagos are lacking.

The White Sea basin provides an excellent opportunity to study stickleback evolution. Since the end of the last glaciation, the West coast of the White Sea experiences isostatic uplift with the current speed of 0.38 cm/year (Kolka and Korsakova 2005). A number of new freshwater lakes forms every century as a result of gradual isolation of former sea bights, whereby shallow bay mouth banks are surfaced by coastline uplift and the salt water connection between the sea and the emerging lake is cut. Initially, the residential population in the new freshwater body evolves in the presence of a flow of genes from anadromous marine fish that use the same lake as a spawning ground. This gene flow facilitates the accumulation of adaptive alleles which are present in marine fish at low frequencies.

We study DIs that exist in ten lacustrine populations of threespine stickleback, located within 120 km of the coastline. All these populations have evolved independently from the marine stickleback population of the White Sea. Examination of the genomic sequences of DIs in multiple populations that have gained the freshwater phenotype in parallel allows us to understand the contributions of individual loci to the adaptation. Analysis of various facets of DI architecture, such as variation of their lengths, frequencies of alleles of marker SNPs across independent populations, and the densities of marker SNPs can improve our understanding of processes that led to their formation.

## Materials and Methods

### Collection of Samples and Ethical Statement

We analyzed ten independent freshwater populations (supplementary table S1, Supplementary Material online) subdivided into two categories: older (>600 years) and young (30–250 years; table 1). The four younger populations and two of the older populations (MAS and LOB) have been analyzed previously (Terekhanova et al. 2014). Two interconnected Kumyazh’i lakes were pooled into one sample (KUM). We also obtained marine individuals from a previously unsampled location (White Sea, WSBS) and pooled them with the two previously analyzed marine samples (Nilma and anadromous individuals from lake Ershovskoye). We collected 8–24 fish from each population, which were then pool-sequenced at the average coverage of 36× (table 1).

**Table 1:** Description of the Locations of Populations Studied

Sample	ID	Description	Geographic Location	Number of Individuals	Number of Reads	Number of Reads Properly Paired	Mean Coverage
White Sea, WSBS	MAR	Marine	66°57.04′N, 33°10.40′E	12	415,169,461	365,058,887	90
Nilma^a^		Marine	66°30.45′N, 33°7.68′E	16			
Ershovskoye^a^ (anadromous)		Marine	66°32.21′N, 33°3.62′E	10			
Lobaneshskoye^a^	LOB	Freshwater, older	66°33.64′N, 33°13.45′E	8	120,452,652	81,595,168	20
Mashinnoye^a^	MAS	Freshwater, older	66°17.74′N, 33°21.82′E	10	92,891,151	64,737,645	16
Canon	CAN	Freshwater, older	66°16.69′N, 34°13.33′E	24	202,051,636	190,057,977	45
Lake Nilma	LN	Freshwater, older	66°49.15′N, 33.09.75′E	16	185,364,807	171,676,383	41
Son	SON	Freshwater, older	66°17.64′N, 34°14.73′E	24	76,682,927	73,382,541	25
Mashinnoye-3	MAS3	Freshwater, older	66°29.85′N, 33°34.41′E	12	44,514,740	41,667,387	15
Kumyazh'i	KUM	Freshwater, older	66°56.24′N, 33°32.63′E	24	156,410,799	147,101,391	35
Ogorodnoye	OG	Freshwater, older	66°56.80′N, 33°21.07′E	20	89,328,329	79,583,953	27
Belaya Guba	BG	Freshwater, older	66°91.08′N, 32°45.83′E	20	69,020,533	64,178,878	22
Voron'ye	VOR	Freshwater, older	66°95.04′N, 32°41.90′E	20	73,677,728	63,567,981	21
Malysh^a^	MAL	Freshwater, 34* *years	66°18.27′N, 33°25.27′E	20	308,787,163	270,452,403	68
Goluboy^a^	GOL	Freshwater, 34* *years	66°17.20′N, 33°23.29′E	20	247,299,313	165,873,385	41
Ershovskoye^a^ (residential)	ER	Freshwater, ∼30* *years	66°32.21′N, 33°3.62′E	12	255,561,219	204,988,298	51
Martsy^a^	MART	Freshwater, ∼250* *years	66°35.95′N, 33°15.35′E	10	126,624,880	77,327,734	19

Note.—The sequencing reads from the three marine populations were pooled together. The populations marked with superscript letter (a) have been analyzed previously (Terekhanova et al. 2014).

Fish were caught by scoop-net or landing-net, anesthetized and euthanized with a tricaine methane sulfonate solution (MS222), and then immediately fixed in 96% ethanol. Fish collection was conducted under the supervision of the Ethics Committee for Animal Research of the Koltzov Institute of Developmental Biology Russian Academy of Sciences.

### Genome Sequencing

Total genomic DNA was extracted from each individual using Wizard genomic DNA purification kit (Promega). Prior to library preparation, DNA samples of between 8 and 24 (table 1) fish from the same population were pooled in equal proportions. Samples from populations OG, BG, VOR, MAS3, and SON were prepared with TruSeq PCR-free protocol (Illumina) and sequenced using HiSeq4000 with 150 bp paired-end reads at Norwegian Sequencing Center (Oslo, Norway). The remaining samples WSBS, KUM, LN, and CAN were prepared according to the TruSeq DNA Sample Preparation Guide (Illumina) and sequenced using HiSeq2000 with 101 bp paired-end reads. Sequencing reads for each population are available at the NCBI Short Read Archive (http://www.ncbi.nlm.nih.gov/sra; last accessed August 21, 2019; accession numbers of the projects are SRP023197 and SRP151980). The reliability of the average freshwater allele frequency estimation from the pool-sequencing of individual populations was assessed and proved in our previous paper (Terekhanova et al. 2014). Here we reanalyze several samples from that paper and use the same protocols for sequencing of the other populations.

### Genome Mapping

The reads were trimmed with trimmomatic version 0.27 (Bolger et al. 2014) and then mapped to the reference genome of the *G. aculeatus* obtained from the UCSC (https://genome.ucsc.edu/; last accessed August 21, 2019) using bwa mem program from the BWA package (Li and Durbin 2009). The alignment was then converted to bam and sorted with the programs from the samtools package (Li 2011). Aligned reads were processed with picard-tools (http://broadinstitute.github.io/picard/; last accessed August 21, 2019) to remove duplicate reads. SNPs were called with the mpileup program of the samtools package (Li 2011).

### Identification of Marker SNPs and DIs

We call position as a “marker SNP” in which marine population has the allele with a frequency below 0.2 and at least one of the ten older freshwater populations have the same allele with a frequency above 0.8 and vice versa.

**Fig. 1.**
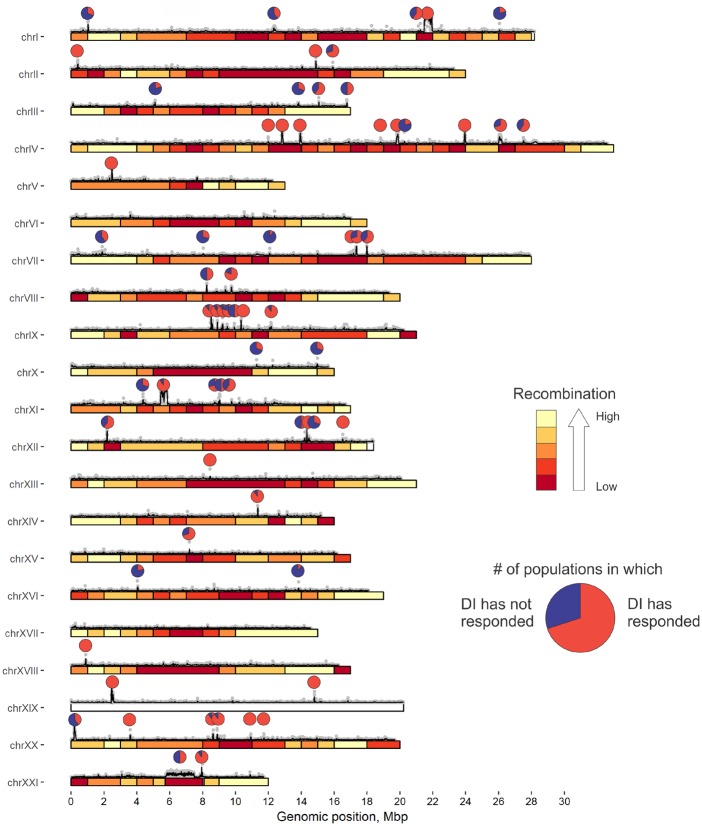
—Genomic positions of DIs. The colors from red to yellow correspond to the five bins of recombination rate from low to high (average values for 1 Mb genomic windows). For each identified DI, the pie chart shows DI pervasiveness, that is, the fraction of the populations (out of ten) in which this DI has “responded,” so that the marker SNPs carry the freshwater alleles at mean frequency of >0.5. Above the bars: gray dots, marker SNP densities in each 5 Kb window; black lines, their smoothing by loess function with the span = 0.0005.

To identify the DIs, we used the ten freshwater populations of older age (table 1). Populations of younger origin were not used as a large portion of their freshwater alleles have not reached high frequency presumably due to insufficient time. We applied the approach similar to that was developed in our previous paper (Terekhanova et al. 2014) that describes well the observed peaks of divergence between marine and freshwater populations (fig. 1). For each population, we first traversed the genome in 10 Kb genomic sliding windows (in 1 Kb steps), listing all windows carrying at least 10 marker SNPs, and calculated the average frequency of the freshwater allele at marker SNPs in those windows. Next, from those windows, we picked the window with the maximum mean freshwater allele frequency; if the freshwater allele frequency in this window was >0.5, this window was then considered the “seed” of a putative population-level DI. We then extended this putative DI along the sequence in each direction by merging it with adjacent 10 Kb windows in 1 Kb steps, until a window was reached in which the mean freshwater allele frequency has declined by more than 30%, compared with the seed window. Then the window with the next highest freshwater allele frequency was used, and the procedure was repeated till no more seeds could be identified outside the already identified putative population-level DIs. After repeating this procedure for all populations, we merged all putative population-level DIs across all populations with lengths of 15 Kb or more if they were located within 30 Kb from each other to obtain the putative list of DIs. For the final list of DIs, we kept only those putative DIs containing at least one 10 Kb window with more than 50 SNPs, each of which was a marker SNP in at least one population. The freshwater allele frequency of a DI at a given population was defined as its mean frequency across the 20% of the windows with the highest freshwater allele frequency within the DI. Finally, to obtain the ultimate list of population-level DIs, we merged all putative population-level DIs within the boundaries of a single DI, irrespective of the distance between them; and discarded those population-level DIs, where the freshwater allele frequency was below 0.5.

We also repeated the results using the above algorithm but keeping the putative DIs containing at least one 10 Kb window with more than 40 marker SNPs (supplementary table S2 and fig. S1, Supplementary Material online); or without merging all putative population-level DIs across all populations (supplementary table S3 and fig. S2, Supplementary Material online); or by mapping the reads to the latest reference stickleback genome (Peichel et al. 2017; supplementary table S4 and fig. S3, Supplementary Material online).

PCA-analysis was performed with R-function prcomp on the input of frequencies of freshwater alleles in marker SNPs located inside DIs. Freshwater allele frequency for each DI was calculated as the mean across the 10 Kb windows with the highest values covered 20% of the DI.

### Fst, Dxy, and π Calculation

We calculated Fst, Dxy, and π values for all 5 Kb nonoverlapping genomic regions. To calculate Fst, we used mpileup2sync.jar and fst-sliding.pl programs from the popoolation2 package (v. 1201) (Kofler et al. 2011). We calculated Dxy and π as the average value across all sites with 1 or 2 alleles. Dxy was calculated at each site as *p*_11_ × *p*_22_ + *p*_12_ × *p*_21_, where *p*_11_ and *p*_12_ are the frequencies of the two alleles in freshwater population and *p*_21_ and *p*_22_ are the frequencies of the same alleles in marine population. π at each site was calculated as 1 − (*p*_1_^2^ + *p*_2_^2^), where *p*_1_ and *p*_2_ are the frequencies of the two alleles.

### Genomic Annotation

Genomic annotation was obtained from the Ensembl database release 72 (Ruffier et al. 2017). We also used BLAST search (BlastX algorithm) against the nr database to annotate some stickleback genes overlapping DIs, using the top BLAST result hit as the homolog if its *e*-value was below 0.01. Gene Ontology (GO) analysis was performed using the R package clusterProfiler (Yu et al. 2012).

### Recombination Rate

We obtained the mean coalescent-based population recombination rates for each 10 Kb window in the G2L freshwater lake population from Feulner et al. (2015) and Feulner PGD (personal communication). These recombination rates are population size-scaled, ρ = 4*N_e_r*, where *r* is the number of expected cross-over events per 10 Kb per generation. For the permutation analyses (see below), the mean *r*-values over all 10 Kb windows were obtained for each 1 Mb window, and the *r*-values were categorized into five bins. Each DI was classified as belonging to one of the recombination categories; if it fell on a boundary between two bins, we recategorized the genomic segment carrying the smaller part of the DI as belonging to the same bin as the larger part of the DI, so that each DI would fall into one bin. When testing clustering of DIs while accounting for the recombination rate variation, we permuted DIs only across the regions belonging to the same bin. Permutation analysis was performed with shuffleBed from the bedtools2 (Quinlan and Hall 2010) with the additional parameter –noOverlapping. *P* values were calculated as the proportions of the1,000 independent permutation trials having values lower than the observed ones.

### Marker SNP Overlap

We calculated marker SNP overlap (*R*) for each DI as follows. We chose those populations possessing at least 20 marker SNPs in the DI. For each of the *N* pairs of such populations (if #populations = 3, then *N *=* *3; #populations = 4, *N *=* *6, etc.), we determined the corresponding sets of marker SNPs, *A* and *B*, that is, numbers of SNPs with the frequency of the freshwater allele >0.8 (see above) in the corresponding two populations from a pair. To avoid misclassifying a shared marker SNP as present in just one of the two compared populations, if it was present in one of the populations with frequency ≥0.8, but in the other, with the frequency between 0.5 and 0.8, it was also considered present in both populations; this correction may bias marker SNP overlap estimates upward. We then calculated marker SNP overlap as the mean, over all *N* pairs of populations, of the ratio of the number of the SNPs shared between *A* and *B* and the lesser of the numbers of SNPs in *A* and *B:*

$$R=\frac{\sum_{i}^{N}\frac{A\cap B}{\min(A,B)}}{N}.$$

The marker SNP overlap for the 25% genomic segments of DIs were calculated similarly, except that we required presence of 5, rather than 20, marker SNPs in a segment in each population from a pair.

The marker SNP overlap of a DI for a particular population (population-level DI) was estimated similarly, except that only those population pairs involving the considered population were used.

## Results

### DIs Are Clustered into Archipelagos

**Fig. 2.**
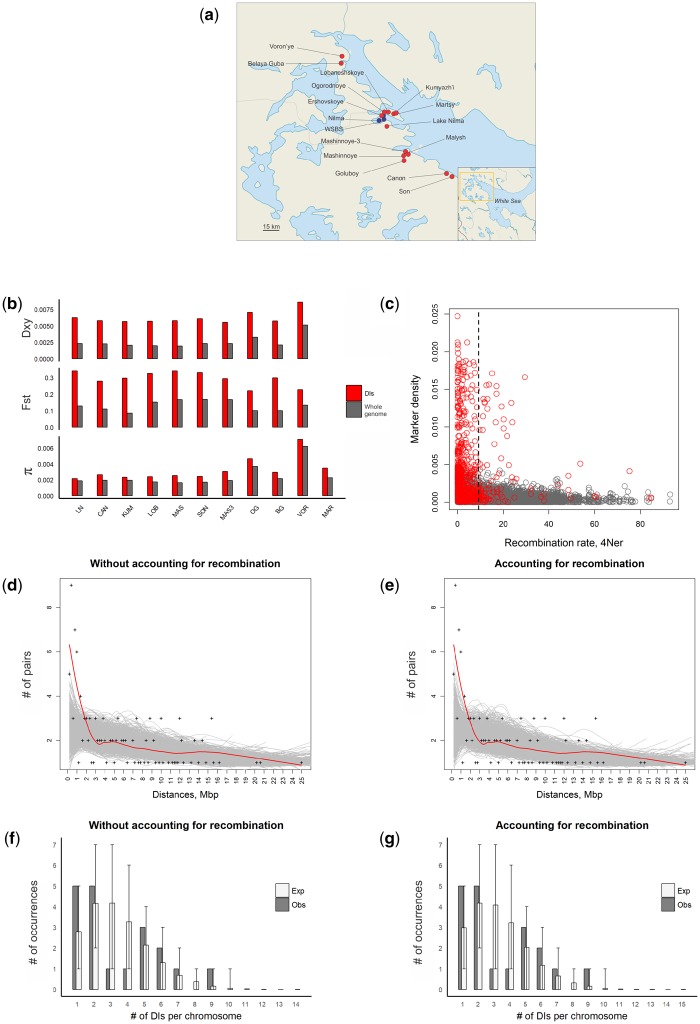
—Divergence in the freshwater populations. (*a*) Map showing the locations of populations studied. (*b*) Dxy and Fst calculated between one marine and ten older freshwater populations; π calculated for one marine and ten older freshwater populations. (*c*) Marker SNP density plotted against recombination rate in 10 Kb genomic windows. Red color denotes windows located inside identified DI regions; dark gray, all other genomic segments. Black dashed line, the average recombination rate across the genome. (*d*–*e*) Distribution of distances between all pairs of DIs located on the same chromosome. Each cross denotes the number of pairs of DIs falling into a particular 200 Kb distance bin (horizontal axis) from each other. Lines, loess smoothing (span = 0.5) for actual data (red) or for each of the 1,000 reshuffling trials (gray) without (*d*) and with (*e*) accounting for local recombination rate. (*f*–*g*) Distribution of the numbers of DIs per chromosome without (*f*) and with (*g*) accounting for the local recombination rate. Expected numbers are calculated from the 1,000 permutation trials.

We performed pooled sequencing of between 8 and 24 individuals from each of the 10 relatively old freshwater populations and of the 4 populations of recent origin (table 1 and fig. 2*a*), 6 of which had been analyzed previously (Terekhanova et al. 2014). We identified a total of 180,249 diallelic SNPs where an allele with a frequency below 0.2 in the ancestral marine population reaches a frequency above 0.8 in at least one of the ten older freshwater populations. 18.6% of these marker SNPs are clustered into 65 DIs (i.e., regions having number of marker SNPs >50 in at least one of its 10 Kb genomic regions, fig. 1 and supplementary table S5, Supplementary Material online, see Materials and Methods). The overall divergence between marine and freshwater populations within these DIs is almost 2.5 times higher than in the rest of the genome (mean Dxy over ten comparisons: 0.0062 within DIs and 0.0026 outside DIs; fig. 2*b*).

The DIs are characterized by reduced rates of recombination: The mean coalescent-based population recombination rates 4*N_e_r* (Feulner et al. 2015) for DIs and for the whole genome are 6.63 and 8.24, respectively. The boundaries of three of the DIs, I-4, XI-2, and XXI-1, match the boundaries of known inversions, in line with previous findings (Jones et al. 2012). However, even within the noninversion DIs, the recombination rate (6.73) is lower than the genome average. Still, some DIs recombine fast: The recombination rates within 11 DIs are above the genome average by factors of up to 7.7 (fig. 2*c* and supplementary table S6, Supplementary Material online).

The DIs are clustered within chromosomes. In all 30 pairs of DIs that occur on the same chromosome, the 2 DIs are located within 1 Mb of one another, although only 10.6 such pairs are expected if the DIs were distributed across the genome randomly (*P* < 0.001, fig. 2*d*). This difference remains significant even when the randomization procedure takes into account the heterogeneity of the recombination rate between genomic regions (30 vs. 11.3, *P* < 0.001, fig. 2*e*). Thus, within-chromosome clustering of DIs cannot be explained by variation of recombination rates. The variance of the numbers of DIs per individual chromosome was also higher than expected if they were randomly distributed, although this difference was less pronounced (2.411 observed vs. 1.879 expected, *P* = 0.041, fig. 2*f*). We see the same pattern when the recombination rate is controlled for (2.411 observed vs. 1.875 expected, *P* = 0.053, fig. 2*g*, see Materials and Methods). Thirty-four of the DIs formed 12 clusters on individual chromosomes, “archipelagos,” having the distance between the closest members <1 Mb (fig. 1).

### Architecture of Adaptation Differs between Freshwater Populations

**Fig. 3.**
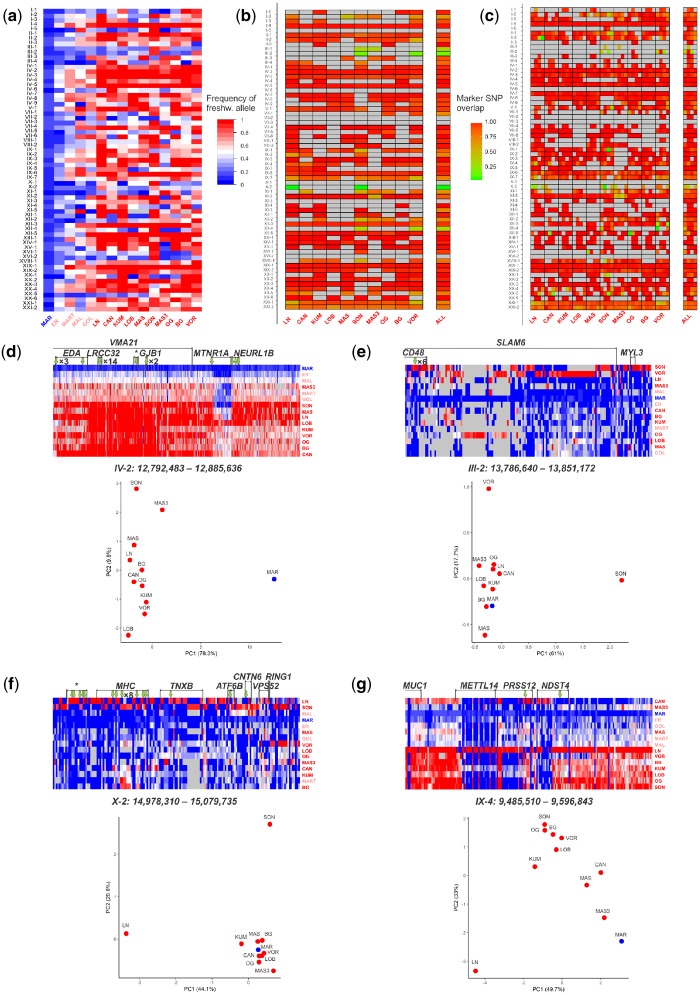
—Genomic architecture of the DIs. (*a*) Freshwater allele frequencies within the 65 identified DIs. Columns, populations from left to right: one marine population, four young freshwater populations, and ten freshwater populations of older ages. Color codes for populations: blue—marine; coral—young freshwater; red—older freshwater. Rows, DIs. Gray cells correspond to values that are missing due to insufficient sequencing coverage. (*b*) DI marker SNP overlap. Columns, individual populations, or the values averaged over all populations (rightmost column); rows, DIs, gray cells, missing data. (*c*) The marker SNP overlap of a DI varies along its length. This panel is similar to panel (*b*), except that each DI is subdivided into four equal bins along its length, and marker SNP overlap is calculated independently for each bin. Only freshwater populations in which this DI had more than five marker SNPs were considered; the remaining cells are colored gray. (*d*–*g*) Allelic composition of individual DIs. Rows, populations. Columns, marker SNPs within the DI, with genes overlapping the DIs indicated with brackets (unannotated genes are marked with an asterisk); green arrows indicate nonsynonymous marker SNPs. Cell color, freshwater allele frequency. Gray cells correspond to values that are missing due to insufficient sequencing coverage. Scatterplots are PCA plots based on densities of marker SNPs in each population. (*d*) High marker SNP overlap (*R *=* *0.99) DI IV-2; many of the marker SNPs are nonsynonymous, including 14 marker SNPs within a short exon of the *LRRC32* gene. (*e*) Low marker SNP overlap (*R *=* *0.17) DI III-2. (*f*) Low marker SNP overlap (*R *=* *0.06) DI X-2. (*g*) Variable marker SNP overlap DI IX-4; most of the marker SNPs are shared between populations at the 5′ part of the DI, but are private to some of the populations at the central part of the DI.

An increase in the frequency of freshwater alleles within a DI in a freshwater population implies adaptation to freshwater. Still, a particular freshwater-specific haplotype is not always present in all freshwater populations. Let us say that a DI has “responded” in a particular freshwater population if the mean frequency of freshwater alleles at marker SNPs across at least the 20% of this DI is above 0.5 (fig. 3*a*). Out of the 65 DIs, 45 (69%) responded in an average population. Twenty-one (32%) DIs responded in every population (hereafter, universal DIs), and 49 (75%) responded in at least half of the populations. Only 2 (3%) DIs responded in just one population, and both of them responded in the SON population (supplementary table S5, Supplementary Material online). We define the pervasiveness of a DI as the proportion of populations in which it responded (fig. 1).

DIs residing within known inversions were pervasive, and responded on average in eight populations (supplementary table S6, Supplementary Material online). Among noninversion DIs, universal DIs differed from the remaining DIs in several respects. They had higher mean frequencies of the freshwater alleles across populations where they responded (0.86 vs. 0.79, two-sided Wilcoxon rank sum test *P* = 0.011), higher density of marker SNPs in the core region shared between populations (see below; 0.0058 vs. 0.0039, two-sided Wilcoxon rank sum test *P* = 0.048), and possibly longer core regions (40.1 vs. 29.4 Kb, two-sided Wilcoxon rank sum test *P* = 0.092).

### Genetic Architecture and Evolutionary History of Individual DIs

Data on multiple freshwater populations allowed us to study the reproducibility of the allelic composition of a DI between populations. In line with the “precast bricks” model (Terekhanova et al. 2014), one can assume that at a particular DI the same freshwater-adapted haplotype was recruited and spread in every freshwater population in which this DI has responded. Alternatively, multiple freshwater-adapted haplotypes could arise at a given DI in the metapopulation of sticklebacks (Bassham et al. 2018). If these haplotypes survived to the present, we may observe that the same DI would have different marker composition in different freshwater populations.

We characterized the diversity of haplotypes within each DI in terms of their allelic composition. In most DIs the sets of marker SNPs that distinguish different freshwater populations from the marine population are similar, suggesting recent common ancestry of the selected alleles in these populations (fig. 3*b* and supplementary table S6, Supplementary Material online). However, several DIs deviate from this pattern to varying degrees. For each DI in each population, we measure the extent to which its marker SNPs overlap those in other freshwater populations (see Materials and Methods for details). We also calculate the average marker SNP overlap over all populations. The mean marker SNP overlap across all DIs is 0.87 (supplementary table S6, Supplementary Material online), implying high consistency of marker SNP composition between populations (fig. 3*b*–*d*). However, for four of the DIs, overlap is below 0.4 (fig. 3*e*–*f* and supplementary table S6, Supplementary Material online). This suggests that some of the DIs originated through recruitment of different haplotypes in different populations. The DIs with extremely low marker SNP overlap are III-2, which shares only 17% of marker SNPs between the two populations that were used to calculate the overlap (fig. 3*e*), and X-2, for which this figure is 6% (fig. 3*f*). Notably, these two DIs overlap genes that are involved in immune response: *CD48* and *SLAM6* in III-2; and *MHC*-associated gene (*mhc1zea*; Kersey et al. 2016) and *CXADR* in X-2. Furthermore, these two DIs possess above average rates of recombination (41.0 and 11.0, respectively, which is higher than the genome average of 8.2).

### Evolutionary History of a DI May Vary along Its Length

We hypothesized that different segments of an individual DI may differ in their evolutionary history. To study this, we have first subdivided each DI into four bins along its length, and analyzed these bins independently. For some of the DIs, we found that the marker SNP overlap varies along their length (fig. 3*c* and *g*). This suggests that even within a single DI, different numbers of freshwater haplotypes are recruited across its segments. The segments with low average marker SNP overlap were also less pervasive (0.53 vs. 0.72 respectively; supplementary fig. S4*a*, Supplementary Material online). Therefore, some of the DIs were likely comprised of a segment resulting from recruitment of the same freshwater haplotype in different freshwater populations, neighbored by a segment where different haplotypes were recruited in different populations (fig. 3*c*). Finally, the low-overlap segments were characterized by higher recombination rates than the remaining segments (9.94 vs. 5.46; supplementary fig. S4*b*, Supplementary Material online). Therefore, the differences between parts of a DI likely result, at least in part, from the underlying recombination structure.

Furthermore, parts of a DI sometimes had radically different values of pervasiveness: Although the markers contained near the center of the DI carried freshwater alleles in all or nearly all freshwater populations, near the DI edges some of the populations were comprised solely of marine alleles (e.g., supplementary fig. S5, Supplementary Material online).

To study this in more detail, we defined population-specific DIs independently for each population and studied the reproducibility of their boundaries between populations. In general, the coordinates of the population-specific DIs matched well between populations, or at least overlapped strongly. The positions of their boundaries were similar: The boundaries of 65% of population-specific DIs where within 50 Kb of the boundaries of the DI defined from all populations; 84%, within 100 Kb; and the rest 16% on the distance within 200 Kb (supplementary table S7, Supplementary Material online). For the three inversion DIs, the positions of the population-specific DIs overlapped by 79% across populations as expected. However, even in some of the DIs not associated with inversions, for example, IV-3 and V-1, the boundaries coincided precisely between some of the populations in which they responded (supplementary fig. S5*a* and *b*, Supplementary Material online). Despite the overall high conservation of the positions of the boundaries, in some population pairs, population-specific DIs comprising the same DI overlap only marginally; DIs II-2 and XVIII-1 are examples of this (supplementary fig. S5*c* and *d*, Supplementary Material online).

For each DI, let us call the segment of the genome which is included in all population-specific DIs its core, and the remainder of the DI its periphery. The length of the periphery is not correlated with the length of the core (ρ = −0.19, *P* = 0.13; supplementary fig. S6, Supplementary Material online). By contrast, it is negatively correlated with the marker SNP overlap within this DI (ρ = −0.59, *P* = 9.0 × 10^−7^). The recombination rate is higher on the periphery compared with the core region (average recombination rate for core and peripheral segments are 5.39 and 7.15, respectively, two-sided Wilcoxon test *P* = 0.016). This could be in part due to variation in the rate of recombination, and the recombination rate is correlated with marker SNP overlap (ρ = −0.41, *P* = 2.3 × 10^−3^) and periphery length (ρ = 0.34, *P* = 8.2 × 10^−3^; supplementary fig. S6, Supplementary Material online).

### Putative Balancing Selection on DIs

Because we focused on marker SNPs with very different frequencies between the marine and the freshwater populations, in the majority of the detected DIs the frequency of the freshwater haplotype in the freshwater populations is high. However, this is not always the case. A striking exception is the DI XXI-1 which coincides with the longest identified inversion (Jones et al. 2012). In this DI, in the marine population, the frequency of freshwater alleles in marker SNPs is very low (∼4%), which is lower than for an average DI (∼11%). In all freshwater populations, the frequencies of freshwater alleles are elevated; however, contrary to what we see in most other DIs, they always remain at an intermediate level and never reach 100% (the mean allele frequency across all freshwater populations: 0.54, range: 0.29–0.81; supplementary table S5, Supplementary Material online). This could be explained by a weaker positive selection on this DI in freshwater populations. However, under moderate selection, we would expect a strong dependence of the freshwater haplotype frequency on the population age (Terekhanova et al. 2014). For DI XXI-1, we see no such dependence. Moreover, in the two very young freshwater populations from our previous study (Terekhanova et al. 2014), the freshwater haplotype frequency in this DI is already rather high (43% and 65% after 30 and 250 years, respectively).

In some other DIs, the freshwater allele frequency is also intermediate and independent of the age of the population (supplementary table S5, Supplementary Material online). As candidate targets of balancing selection, we have selected the DIs with the lowest difference in freshwater allele frequencies between the young and older freshwater populations. The first four DIs on the list were IV-8, XXI-1, IX-7, and IV-4. The first three of these, IV-8, XXI-1, and IX-7, overlap multiple genes involved in the immune system: DI IV-8 overlapped with *HSPA9* and positioned within 15 Kb of the *IGBP1* and *MAGT1*; the large inversion DI XXI-1 overlapped with *RBCK1*, *SOCS6*, *CD226*, *RRS1*; and DI IX-7 overlapped with *CLEC6A*, *CD209*, and *UNC93B1*. The fourth DI IV-4 overlapped a pair of duplicated *AKR1B1* genes involved in reproduction (supplementary table S8, Supplementary Material online).

### Positive Selection outside DIs

Previously, we have shown that marker SNPs are enriched in nonsynonymous substitutions compared with the nonmarker SNPs segregating within the marine population (Terekhanova et al. 2014), and interpreted this as a sign of positive selection. Here we analyze marker SNPs using data on many populations. In line with our previous results (Terekhanova et al. 2014), we find that the d*N*/d*S* ratio for marker SNPs outside DIs is higher than that for marker SNPs within DIs, signifying high prevalence of positive selection outside DIs and/or less efficient selection in DIs due to lower recombination rate and thus stronger genetic drift in them (supplementary fig. S7, Supplementary Material online). Notably, the d*N*/d*S* ratio for marker SNPs is higher for those marker SNPs that are present in at least five populations, compared with all other marker SNPs, both inside the DIs (0.301 vs. 0.243) and outside them (0.615 vs. 0.315; supplementary fig. S7, Supplementary Material online). This suggests that the fraction of positively selected marker SNPs is the highest among those marker SNPs at which the freshwater allele is present in many populations.

## Discussion

The high speed of adaptation of threespine stickleback populations to the freshwater environment is made possible by the fact that the freshwater alleles are present at low frequencies in the ancestral marine population (Colosimo et al. 2005; Schluter and Conte 2009). Adaptation to such a radically different environment is likely to be genetically complex and to involve many loci, as was shown for other species (Renaut et al. 2013; Gao et al. 2017). Identifying all loci responsible for a complex adaptation is a difficult task (Hoban et al. 2016). However, in threespine sticklebacks, similar to other species that have adapted to widely different environments (Jones et al. 2012; Nadeau et al. 2012; Renaut et al. 2013; Sodeland et al. 2016), some of the loci responsible for adaptation are located in DIs—regions of elevated divergence between the freshwater and marine populations (Turner et al. 2005; Feder and Nosil 2010). We do not know what proportion of adaptive differences between the marine and freshwater populations of threespine stickleback are confined to DIs, although this proportion is likely to be high (Terekhanova et al. 2014).

DIs are scattered throughout the genome, and are relatively easy to identify as sufficiently long regions with an increased density of marker SNPs—sites where marine and freshwater populations carry different common alleles. It is not clear what factors promote DIs formation and are responsible for variation in their lengths. A relatively long DI may arise due to multiple targets of positive selection located within a relatively short genomic region, to very strong selection acting on just a single target (Feder et al. 2012; Flaxman et al. 2013), and/or to locally reduced recombination rate (Feulner et al. 2015; Samuk et al. 2017).

To elucidate the processes involved in DIs formation, we studied ten independent freshwater populations of threespine stickleback which originated recently in the basin of the White Sea. We found that DIs tend to reside in genomic regions of low recombination rate, in line with the previous observations (Samuk et al. 2017), probably because reduced recombination facilitates their formation (Barton 2000; Yeaman et al. 2016). This may seem paradoxical because recombination usually facilitates adaptation creating new combinations of alleles (Felsenstein 1974). However, low recombination rate also makes adaptation easier to detect by increasing the length of a DI which emerges as a result of positive selection acting on an individual target (Jones et al. 2012; Nadeau et al. 2012; Renaut et al. 2013; Sodeland et al. 2016). Reduced recombination is not a necessary condition for the formation of DIs: around some of them, recombination is high (fig. 2*c* and supplementary table S6, Supplementary Material online).

DIs also tend to be clustered along chromosomes, and this effect cannot be explained by differences in the recombination rate (fig. 2*d*–*g*). Similar archipelagos of DIs were previously observed in the Atlantic cod (Bradbury et al. 2013) and cichlid species (Malinsky et al. 2015); DIs also seem to be clustered in stick-insects (Riesch et al. 2017) and munias (Stryjewski and Sorenson 2017), although no statistical analyses were performed to test this. In human populations, some of the genomic regions that likely harbored selective sweeps, as defined by the iHS scan, are also clustered along the chromosomes, and this clustering can be only partially explained by variation in recombination rate, gene density, or background selection (Johnson and Voight 2018).

Clustering of DIs may occur because neighboring DIs facilitate formation of each other, for example, due to the process of divergence hitchhiking. This process increases the probability of fixation of a new beneficial mutation located near another beneficial mutation (Via 2009; Feder et al. 2012), thus expanding a DI or producing an archipelago of DIs. As a result, when two incompletely isolated populations adapt to different environments, the locally adaptive alleles tend to reside in tightly linked loci, forming long haplotype blocks (Yeaman and Whitlock 2011; Yeaman et al. 2016).

Under divergence hitchhiking, one may also expect to see similar frequencies and positive Linkage Disequilibrium (LD) between freshwater alleles at adjacent DIs. However, this prediction of the model is not confirmed by our data: Frequencies of freshwater alleles in nearby DIs are no more similar than in remote DIs (fig. 3*a* and supplementary tables S5 and S9, Supplementary Material online). Similarly, in previous studies, positive LD was observed only for a few of the adjacent DIs (Hohenlohe et al. 2012); and DI divergence and length were found to be independent of the age of the locally adapted population (in the range of thousands of years) (Feulner et al. 2015). This discrepancy is perhaps not surprising. Although the attraction of the DIs may be manifested at timescales of DIs lifespan which may cover millions of years (Nelson and Cresko 2018), its signal may be too weak to be detected at timescales of individual populations which are only thousands of years old.

The number of DIs responsible for the adaptation of threespine stickleback to freshwater that have been detected throughout its range is in the high tens (Hohenlohe et al. 2010; Jones et al. 2012; Terekhanova et al. 2014). Although the set of DIs is far from being identical across populations, often some of these DIs are reused by freshwater populations of independent origin. It seems plausible that some of the DIs are particularly important for adaptation, and they can be expected to be more pervasive. Indeed, pervasive DIs possess greater density of marker SNPs, carry freshwater alleles at higher frequencies in freshwater populations, and probably have longer core region shared between populations (supplementary fig. S6 and table S6, Supplementary Material online). Interestingly, the frequency of the freshwater allele in pervasive DIs tends to be higher than in other DIs even in the marine populations (fig. 3*a*), suggesting that the selection against the freshwater alleles in the marine environment at such DIs can be weaker. The elevated frequencies of freshwater alleles in the pervasive DIs in the ancestral marine population can facilitate their frequent fixation in freshwater populations. Indeed, the frequencies of freshwater alleles in pervasive DIs are higher than in other DIs even in the youngest freshwater populations (correlations between pervasiveness and freshwater allele frequency: lake Ershovskoye [ER], 30 years old [Terekhanova et al. 2014], ρ = 0.58, *P* = 4.33 × 10^−7^; lake Martzi [MART], 250 years old [Terekhanova et al. 2014], ρ = 0.63, *P* = 1.42 × 10^−8;^fig. 3*a*).

Although the average marker SNP overlap of a DI is high, it is below 0.4 for four of them. This suggests that multiple haplotypes were involved in adaptation at a single DI (Bassham et al. 2018). This could be the case under two different scenarios. Exactly the same beneficial allele can arise against multiple backgrounds. Alternatively, selection at different populations could increase the frequencies of different, although probably functionally similar, beneficial alleles (Hermisson and Pennings 2005). Because we are unable to precisely identify the targets of positive selection in our DIs, we cannot say, for a DI with low marker SNP overlap, which scenario has led to its origin. Still, the DIs with low marker SNP overlap usually harbor some proportion of common marker SNPs: Even the DI where this overlap is the lowest shares 6% of the marker SNPs between the only two populations in which it has responded (DI X-2, fig. 3*f*). Therefore, we cannot reject the simplest hypothesis that the beneficial allele involved in adaptation in a DI has been exactly the same in all populations. Some of the DIs with the lowest marker SNP overlap are characterized by above average recombination rates, implying that they also have elevated local effective population sizes (*N_e_*) (Gossmann et al. 2011), possibly because they possess genes in which diversity and recombination are beneficial, such as immune and signaling pathways genes (The International HapMap Consortium 2007; Choi et al. 2016). The presence of multiple haplotypes, with traces of recombination between them, in regions of increased divergence among multiple populations has recently been observed in munia species (Stryjewski and Sorenson 2017). We find that the architecture of a DI may vary along its length (fig. 3*c* and supplementary fig. S4, Supplementary Material online), implying that different haplotypes could have contributed to the formation of even a single DI.

According to our criterion for identification of DIs (see Materials and Methods), coordinates of a DI in individual populations do not need to overlap. However, we find that usually these coordinates overlap substantially, so that a DI possesses a long-shared core region. We also see that the length of the periphery of a DI sometimes varies only slightly. Because most DIs are old, this implies that recombination within a DI may be constrained. Such a constraint could arise due to strong divergent selection and/or to structural variation. The high conservation of DI boundaries over millions of years of their evolution is in line with the theoretical prediction that DIs should accumulate genomic rearrangements that maintain their lengths (Yeaman 2013). Indeed, three of the analyzed DIs reside within inversions which impede recombination (Jones et al. 2012). Linkage disequilibrium is also increased inside the noninversion DIs (fig. 3*d*), which promotes stability of their boundaries, and even between some of them (Hohenlohe et al. 2012). Still, some DIs have only short core regions or even do not overlap at all (supplementary fig. S5, Supplementary Material online).

In general, selection acting at a DI is strong: The average frequency of freshwater alleles across the responded DIs is 0.81 (supplementary table S6, Supplementary Material online). However, in some cases, we observe that the freshwater alleles at a DI only reach intermediate frequencies. This could be due to two reasons: The selection in favor of the freshwater alleles at this DI is weak, so that they have had insufficient time to reach a high frequency; or the equilibrium allele frequency is <1 due to the action of balancing selection. The first explanation for our observations is unlikely to be responsible for the majority of observations, because the selection coefficients for favorable alleles at DIs are usually very high and because the freshwater allele frequency at these DIs is independent of the age of the population (Barrett et al. 2008; Terekhanova et al. 2014). By exclusion, we are left with the second scenario, although it is difficult to test with our data. Individual genes, especially those involved in immune response, may experience balancing selection even within a single habitat due to mechanisms such as heterozygote advantage, frequency-dependent selection, or fluctuating selection. The action of balancing selection in the evolution of immune genes is thought to be the result of host-parasite interrelations (Eizaguirre et al. 2012).

The top candidate for balancing selection is DI XXI-1. This DI is located within the longest inversion and carries an unusually high density of marker SNPs (Terekhanova et al. 2014), suggesting its old age at the level of the metapopulation, and it was found to be one of the oldest among all DIs (∼8 Ma; Nelson and Cresko 2018). Intermediate frequency of the freshwater haplotype and independence of this frequency of population age (fig. 3*a*) suggests that this DI could have experienced balancing selection. Also the selection involved could be negative frequency-dependent, as reported recently for the major histocompatibility complex class IIβ (MHC IIβ) genes in stream-lake stickleback populations (Bolnick and Stutz 2017).

Data on a number of adjacent freshwater populations of independent and relatively recent origin from the White Sea region, reported in this study, complement those on much older populations from the Pacific basin (Bell and Foster 1994; Colosimo et al. 2005; Hohenlohe et al. 2010; Jones et al. 2012). We developed approaches to analysis of the genomic variation based on pool-sequencing data which could be broadly useful for studying the genetic basis of adaptation. The patterns observed in stickleback may contribute to the knowledge of how genomic islands of divergence emerge and how they are involved in speciation in a variety of species. In contrast to the well-studied Pacific stickleback populations, the White Sea area provides a plethora of young lakes, which makes it possible to study the early stages of evolution of freshwater populations, including parallelisms in the genetics of their adaptation. In the future, it will be interesting to investigate functional genomics of the early stages of the sea—lake ecotype transition in the emerging freshwater ecosystems.

## Supplementary Material

Supplementary data are available at *Genome Biology and Evolution* online.

## Supplementary Material

evz175_Supplementary_Data

## Notes

**Data deposition:** Sequencing reads for each population have been deposited at the NCBI Short Read Archive (http://www.ncbi.nlm.nih.gov/sra; last accessed August 21, 2019) under project accession numbers SRP023197 and SRP151980.
