# Supplementary material for: Architecture of Parallel Adaptation in Ten Lacustrine Threespine Stickleback Populations from the White Sea Area
Source: Genome Biol Evol. 2019 Aug 12;11(9):2605–18. doi: 10.1093/gbe/evz175 (PMC6761963; doi:10.1093/gbe/evz175)
Supplement: evz175_Supplementary_Data [file evz175_supplementary_data.zip › Supporting_Information.pdf]

Supplementary Material

Figure S1. Marker SNP overlap (a) and distribution across chromosomes (b-c) of DIs obtained by using developed method, but keeping the putative DIs containing at least one 10 Kb window with more than 40 marker SNPs (instead of 50 as in the main text). Notations as in figs. 2d, 2e and 3b.

Figure S2. Marker SNP overlap (a) and distribution across chromosomes (b-c) of DIs obtained by using developed method, but without merging all putative population-level DIs across all populations. Notations as in figs. 2d, 2e and 3b.

Figure S3. Marker SNP overlap (a) and distribution across chromosomes (b) of DIs obtained by mapping the reads to the latest reference stickleback genome (Peichel et al., Journal of Heredity 2017). Notations as in figs. 2d and 3b.

Figure S4. Rigidity vs. pervasiveness (a) and recombination (b), calculated for 25% segments of DIs.

Figure S5. Different haplotypes constitute DIs IV-3, V-1, II-2 and XVIII-1.

Figure S6. Pairwise scatterplots of different characteristics of the 65 DIs. Black squared frames denote scatterplots with significant Spearman correlations ($P < 0.05$); red line – Pearson correlation.

Figure S7. dN/dS ratio for marker SNPs. dN/dS ratios calculated for marker SNPs which are found in 2-5 freshwater populations (left bar in each pair) or in the 6-10 freshwater populations (right bar in each pair). Red bars correspond to the ratios calculated for marker SNPs located in DIs, and purple bars, to the ratios calculated for marker SNPs located outside of the DIs. Confidence intervals were obtained from the 1,000 independent permutation trials.

Table S1. Fst calculated for all pairs of 10 older freshwater populations studied.

Table S2. Boundaries of population-specific DIs in each of the 10 freshwater populations obtained by using developed method, but keeping the putative DIs containing at least one 10 Kb window with more than 40 marker SNPs. Absent cells correspond to the DIs with freshwater allele frequency < 0.5.

Table S3. Boundaries of population-specific DIs in each of the 10 freshwater populations obtained by using developed method, but without merging all putative population-level DIs across all populations. Absent cells correspond to the DIs with freshwater allele frequency < 0.5.

Table S4. Boundaries of population-specific DIs in each of the 10 freshwater populations, obtained by mapping the reads to the latest reference stickleback genome. Absent cells correspond to the DIs with freshwater allele frequency < 0.5.

Table S5. List of DIs with corresponding freshwater allele frequencies in the populations studied.

Table S6. Properties of the 65 identified DIs. Freshwater allele frequency is calculated as a mean frequency among populations in which DI has responded. Pervasiveness corresponds to the fraction of populations in which the DI has reached freshwater allele frequency > 0.5. Core region length is not defined for DIs VII-3 and XVI-2, which have responded in only one population.

Table S7. Boundaries of population-specific DIs in each of the 10 freshwater populations. Absent cells correspond to the DIs with freshwater allele frequency < 0.5.

Table S8. Genes overlapping DIs or positioned within 15 Kb of them.

Table S9. Differences in allele frequency for pairs of remote DIs (located on distance > 1Mb, same chromosome) are usually no greater than allele frequency differences for pairs of nearby DIs (located <1Mb). First and second rows, averages for absolute values of allele frequency differences. P-values obtained by applying one-sided Wilcoxon test.

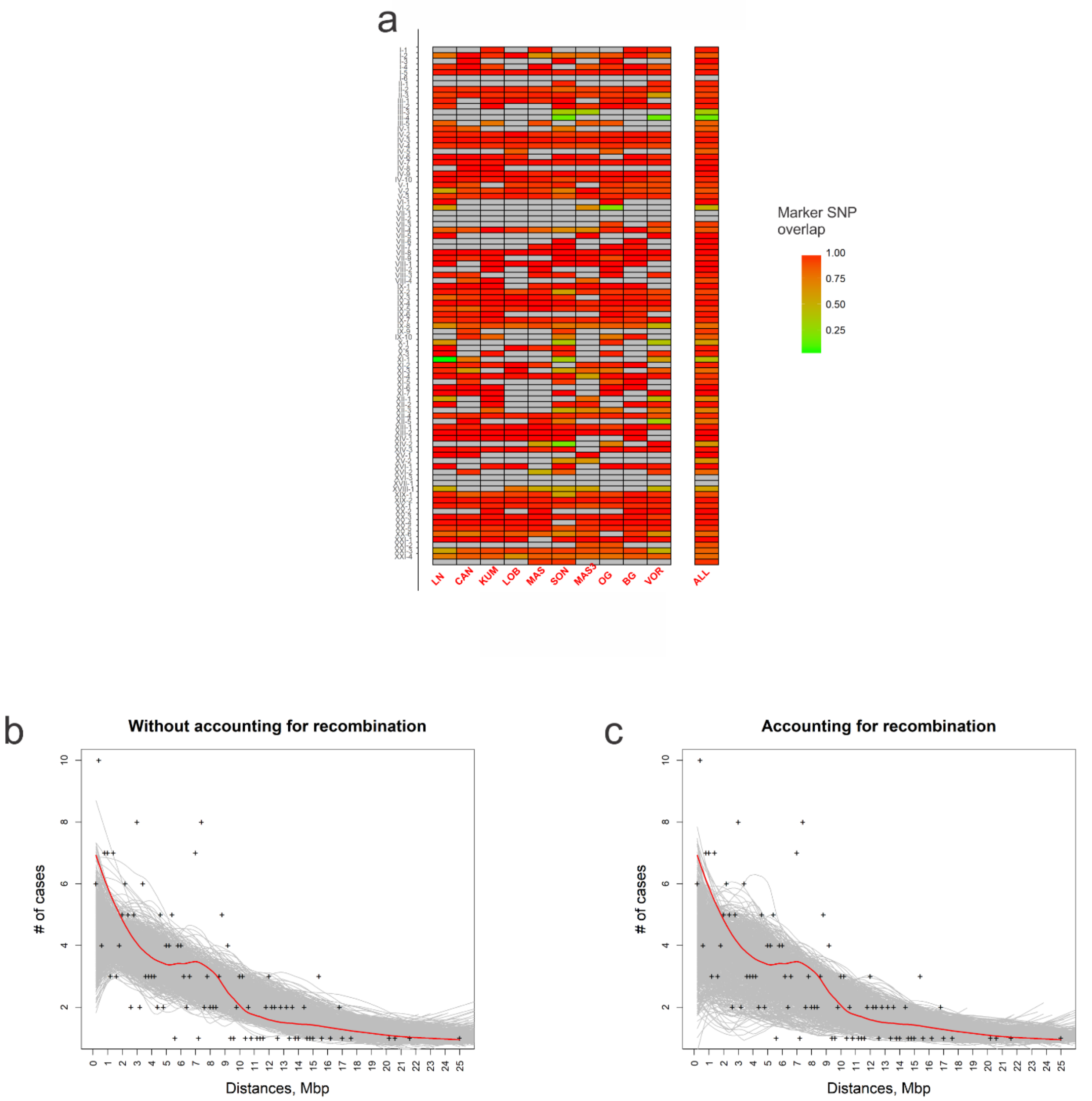

Figure S1. Marker SNP overlap (a) and distribution across chromosomes (b-c) of DIs obtained by using developed method, but keeping the putative DIs containing at least one 10 Kb window with more than 40 marker SNPs (instead of 50 as in the main text). Notations as in figs. 2d, 2e and 3b.

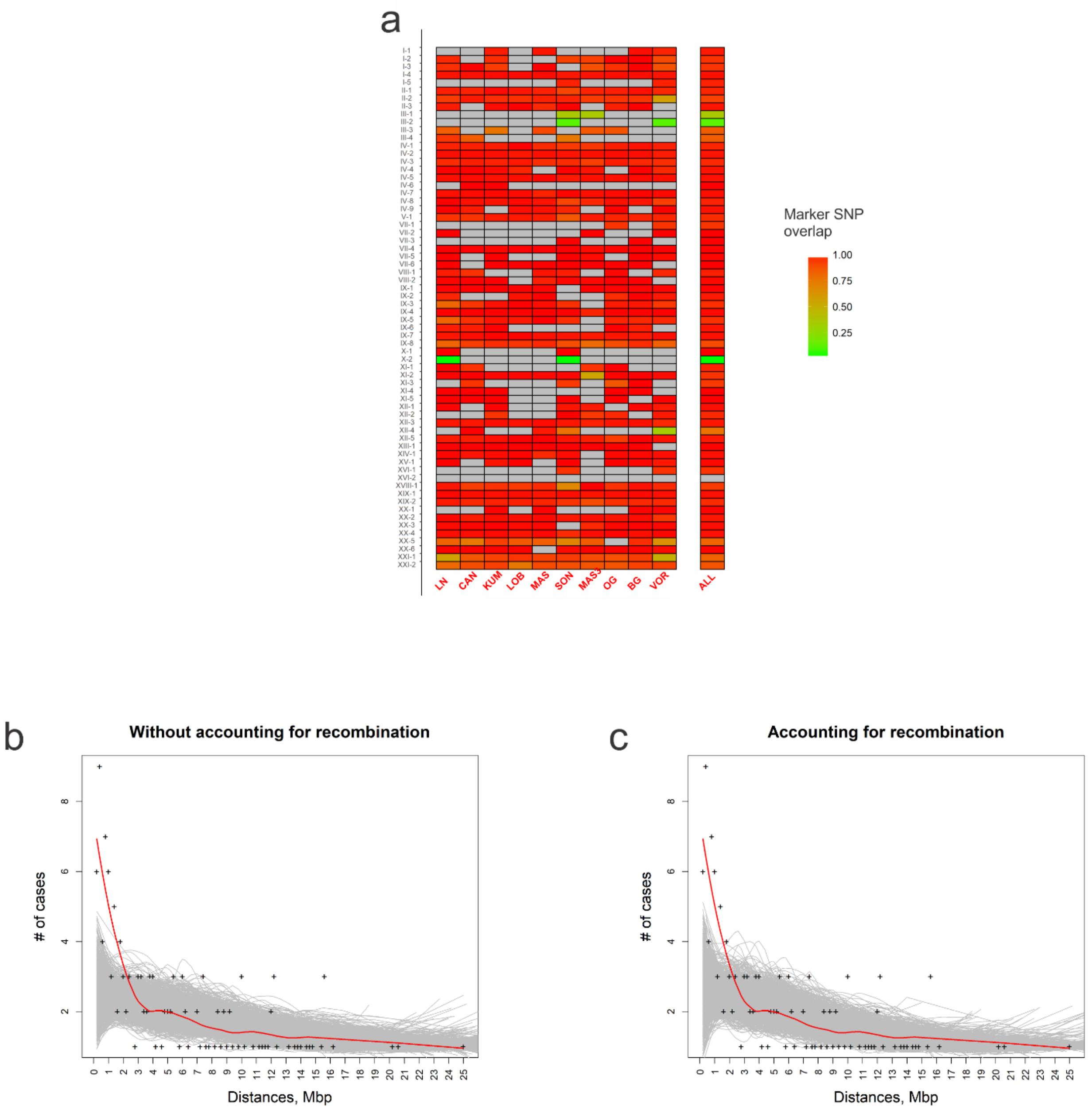

Figure S2. Marker SNP overlap (a) and distribution across chromosomes (b-c) of DIs obtained by using developed method, but without merging all putative population-level DIs across all populations. Notations as in figs. 2d, 2e and 3b.

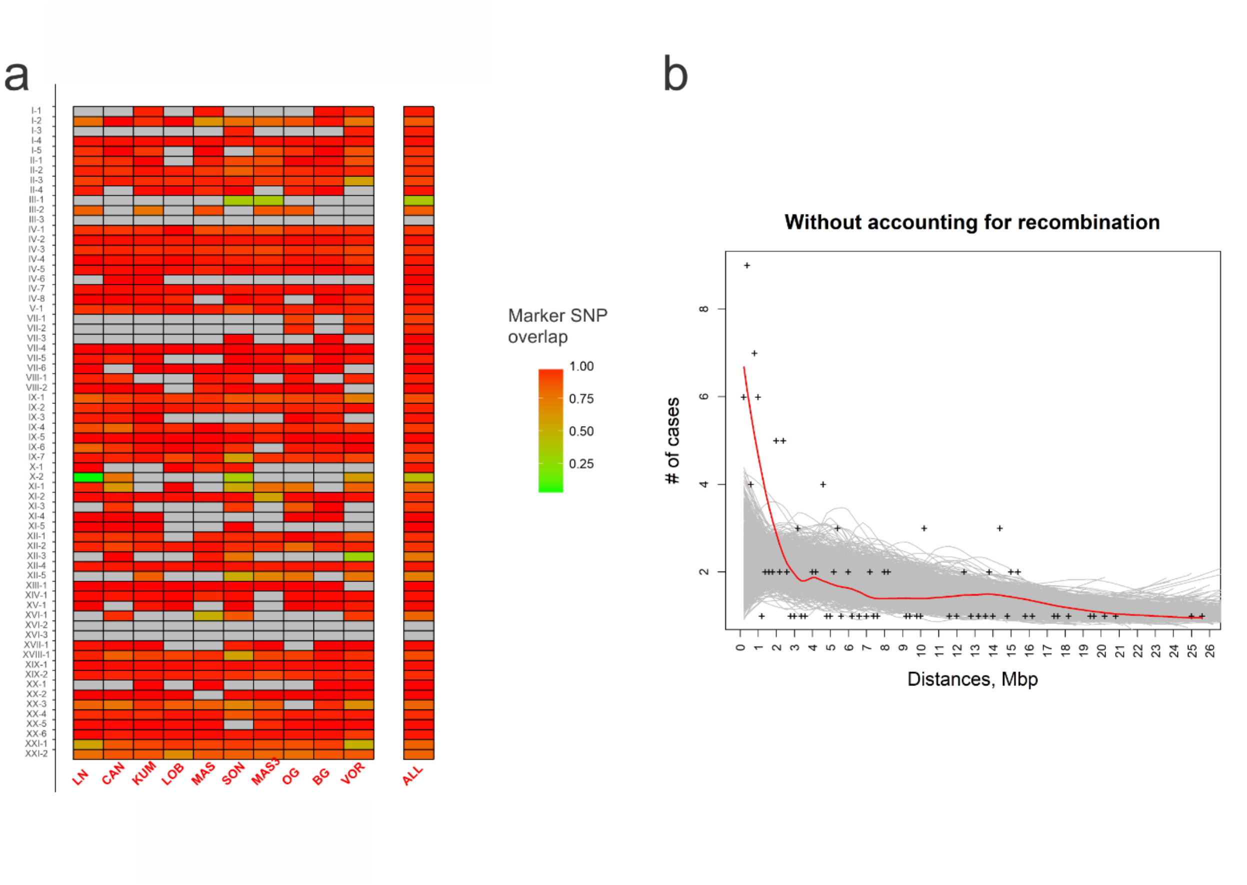

Figure S3. Marker SNP overlap (a) and distribution across chromosomes (b) of DIs obtained by mapping the reads to the latest reference stickleback genome (Peichel et al., Journal of Heredity 2017). Notations as in figs. 2d and 3b.

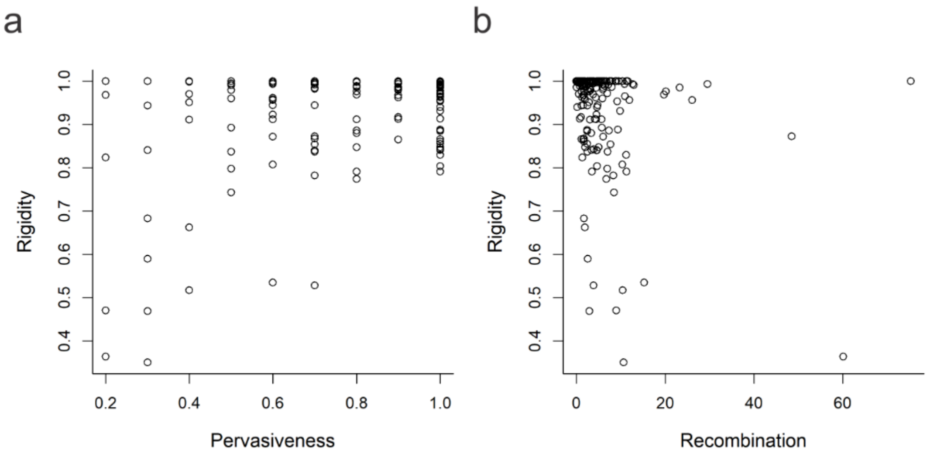

Figure S4. Rigidity vs. pervasiveness (a) and recombination (b), calculated for 25% segments of DIs.

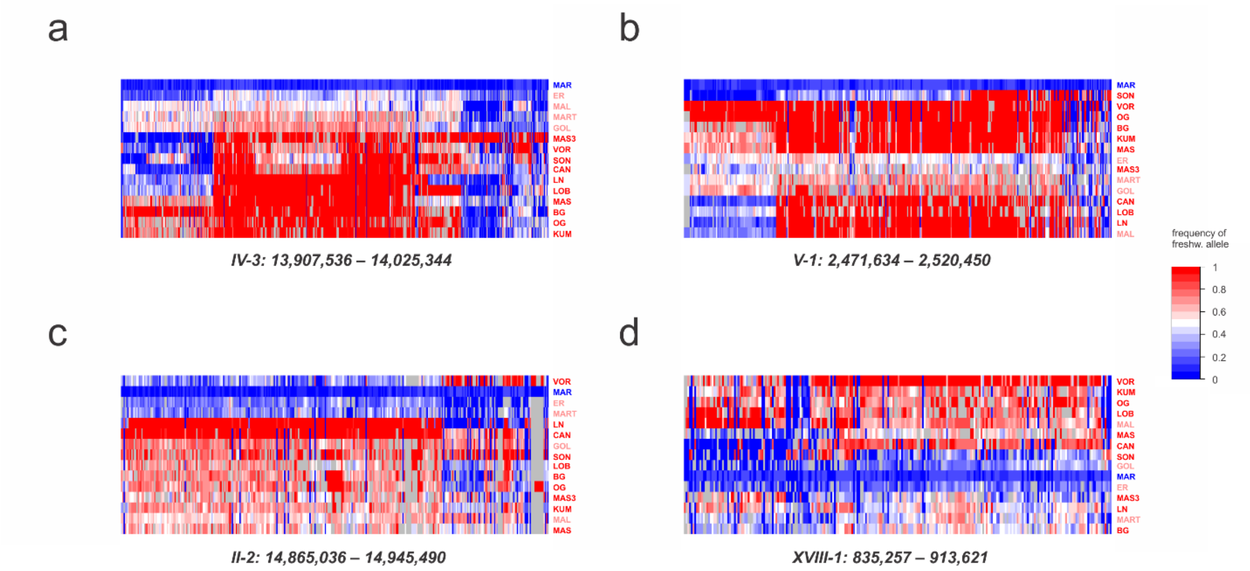

Figure S5. Different haplotypes constitute DIs IV-3, V-1, II-2 and XVIII-1.

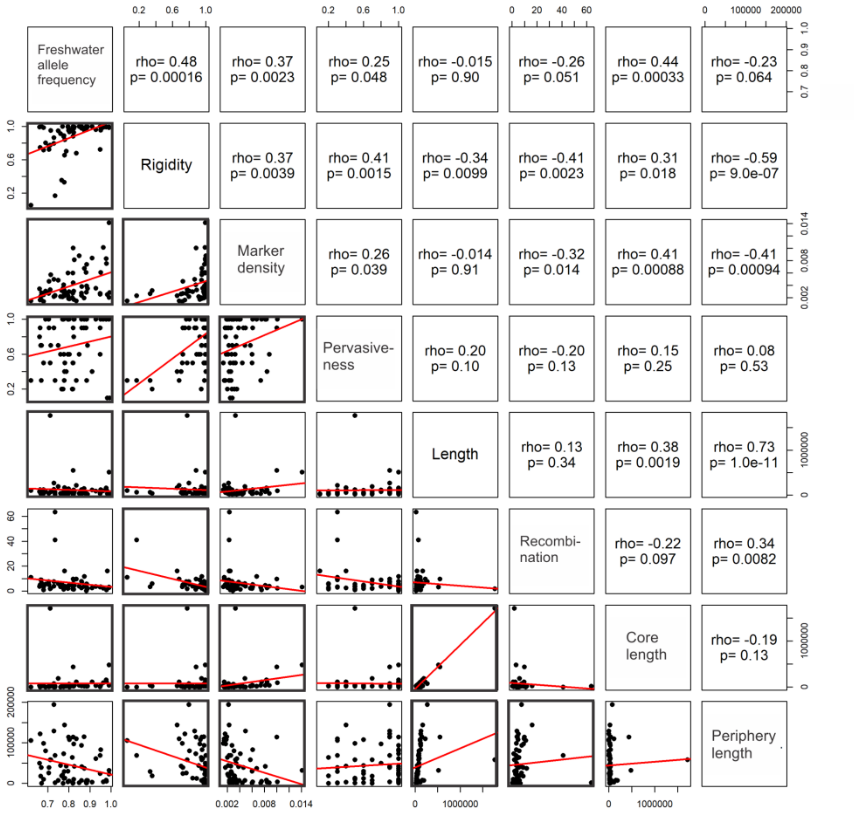

Figure S6. Pairwise scatterplots of different characteristics of the 65 DIs. Black squared frames denote scatterplots with significant Spearman correlations (P < 0.05); red line – Pearson correlation.

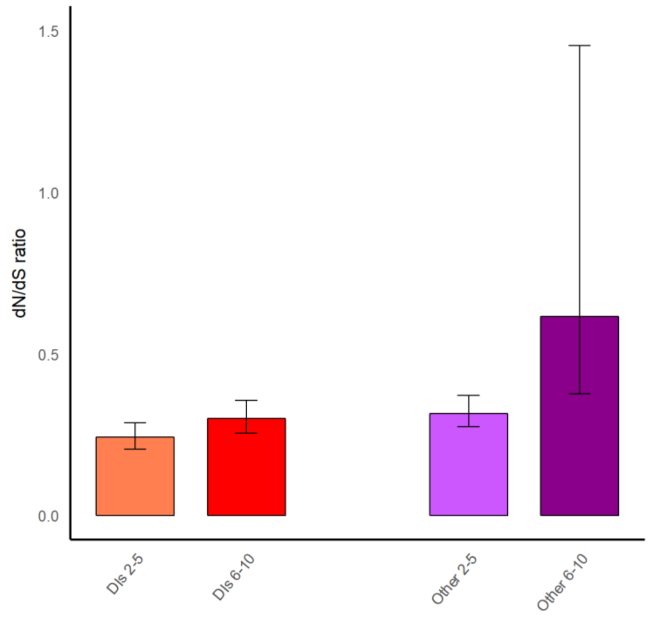

Figure S7. dN/dS ratio for marker SNPs. dN/dS ratios calculated for marker SNPs which are found in 2-5 freshwater populations (left bar in each pair) or in the 6-10 freshwater populations (right bar in each pair). Red bars correspond to the ratios calculated for marker SNPs located in DIs, and purple bars, to the ratios calculated for marker SNPs located outside of the DIs. Confidence intervals were obtained from the 1,000 independent permutation trials.

	BG	CAN	KUM	MAS3	LN	OGO	SON	VOR	LOB	MAS
BG		0.128	0.100	0.164	0.143	0.103	0.171	0.138	0.150	0.161
CAN			0.126	0.195	0.170	0.113	0.202	0.148	0.188	0.204
KUM				0.176	0.135	0.098	0.176	0.137	0.154	0.171
MAS3					0.201	0.157	0.219	0.176	0.202	0.205
LN						0.121	0.222	0.157	0.196	0.210
OGO							0.140	0.089	0.154	0.170
SON								0.168	0.231	0.245
VOR									0.194	0.198
LOB										0.179
MAS										

Table S1. Fst calculated for all pairs of 10 older freshwater populations studied.

DI	Start	End	LN	CAN	KUM	LOB	MAS	SON	MAS3	OG	BG	VOR	MAR
I-1	1,018,000	1,054,000	0.14	0.46	0.81	0.17	0.42	0.09	0.02	0.17	0.94	0.91	0.06
I-2	12,303,000	12,469,000	0.87	0.36	0.47	0.42	0.49	0.86	0.48	0.57	0.43	0.82	0.12
I-3	21,213,000	21,257,000	0.91	0.66	0.78	0.28	0.45	0.37	0.45	0.63	0.68	0.91	0.12
I-4	21,478,000	21,993,000	1.00	0.99	0.99	0.99	1.00	0.99	0.99	0.98	0.99	0.98	0.08
I-5	26,042,000	26,064,000	0.31	0.10	0.24	0.12	0.44	0.95	0.05	0.18	0.43	0.69	0.02
II-1	371,000	517,000	0.83	0.86	0.72	0.59	0.83	0.93	0.87	0.75	0.82	0.78	0.11
II-2	14,865,000	14,947,000	0.98	0.97	0.69	0.70	0.64	0.84	0.62	0.75	0.74	0.80	0.11
II-3	15,909,000	15,930,000	0.65	0.42	0.90	0.52	0.66	0.53	0.40	0.82	0.60	0.05	0.14
III-1	5,088,000	5,126,000	0.14	0.31	0.24	0.16	0.16	0.89	0.64	0.25	0.23	0.22	0.08
III-2	13,784,000	13,853,000	0.40	0.29	0.33	0.32	0.26	0.76	0.25	0.68	0.16	0.76	0.09
III-3	15,061,000	15,089,000	0.74	0.34	0.62	0.34	0.95	0.14	0.91	0.89	0.47	0.54	0.15
III-4	16,764,000	16,793,000	0.77	0.93	0.43	0.23	0.25	0.65	0.85	0.40	0.04	0.70	0.05
IV-1	11,980,000	12,011,000	0.88	0.82	0.92	0.63	0.66	0.89	0.65	0.88	0.97	0.92	0.13
IV-2	12,791,000	12,889,000	0.99	1.00	0.96	0.98	0.99	0.99	0.83	0.95	0.98	0.95	0.12
IV-3	13,905,000	14,027,000	0.98	0.92	0.98	0.98	0.98	0.89	0.97	0.96	0.98	0.85	0.13
IV-4	18,814,000	18,851,000	0.98	0.71	0.90	0.70	0.63	0.63	0.59	0.76	0.91	0.87	0.11
IV-5	19,803,000	19,923,000	0.98	0.99	0.98	0.99	0.98	0.99	0.99	0.98	0.98	0.93	0.16
IV-6	20,265,000	20,288,000	0.23	0.94	0.61	0.43	0.31	0.40	0.15	0.40	0.45	0.22	0.04
IV-7	23,909,000	23,991,000	0.97	0.85	0.72	0.99	0.89	0.64	0.95	0.98	0.67	0.60	0.15
IV-8	26,001,000	26,194,000	0.42	0.84	0.73	1.00	0.69	0.33	0.57	0.74	0.68	0.48	0.17
IV-9	27,497,000	27,605,000	0.56	0.48	0.40	0.94	0.91	0.42	0.34	0.97	0.54	0.89	0.13
V-1	2,466,000	2,521,000	0.94	0.93	0.92	0.90	0.93	0.69	0.52	0.99	0.93	0.99	0.13
VII-1	1,869,000	1,928,000	0.74	0.28	0.25	0.12	0.30	0.87	0.18	0.83	0.37	0.71	0.06
VII-2	8,038,000	8,060,000	0.83	0.02	0.26	0.46	0.12	0.04	0.52	0.45	0.30	0.85	0.14
VII-3	12,052,000	12,088,000	0.16	0.31	0.26	0.29	0.39	0.98	0.17	0.30	0.41	0.41	0.14
VII-4	17,156,000	17,186,000	0.94	0.95	0.97	0.61	0.85	0.87	0.97	0.95	0.58	0.90	0.16
VII-5	17,306,000	17,382,000	0.76	0.58	0.68	0.32	0.41	0.58	0.89	0.58	0.41	0.93	0.15
VII-6	17,970,000	18,012,000	0.72	0.17	0.93	0.48	1.00	0.98	0.37	0.97	0.50	0.36	0.09
VIII-1	8,234,000	8,269,000	0.90	0.40	0.43	0.39	0.79	0.94	0.19	0.58	0.48	0.89	0.12
VIII-2	9,714,000	9,795,000	1.00	0.45	0.57	0.33	0.51	0.78	0.82	0.53	0.54	0.54	0.12
IX-1	8,477,000	8,606,000	0.98	0.99	0.60	0.66	0.75	0.52	0.31	0.69	0.61	0.75	0.12
IX-2	8,884,000	8,931,000	0.95	0.85	0.63	0.51	0.64	0.96	0.38	0.63	0.60	0.56	0.14
IX-3	9,199,000	9,285,000	1.00	0.72	1.00	0.92	1.00	0.99	0.45	0.98	0.97	0.95	0.13

IX-4	9,485,000	9,600,000	0.98	0.55	0.80	0.79	0.48	0.93	0.46	0.91	0.78	0.84	0.12
IX-5	9,901,000	9,940,000	0.75	0.77	0.79	0.07	0.19	0.48	0.35	0.50	0.58	0.11	0.05
IX-6	10,271,000	10,395,000	0.97	0.91	0.68	0.88	0.94	0.90	0.79	0.91	0.93	0.70	0.09
IX-7	12,155,000	12,277,000	0.44	0.85	0.71	0.62	0.56	0.61	0.62	0.76	0.69	0.54	0.09
X-1	11,270,000	11,330,000	0.55	0.52	0.10	0.42	0.48	0.94	0.10	0.30	0.28	0.19	0.06
X-2	14,974,000	15,080,000	0.62	0.28	0.27	0.18	0.14	0.69	0.30	0.41	0.23	0.54	0.10
XI-1	4,275,000	4,497,000	0.29	0.39	0.22	0.20	0.11	0.29	0.89	0.77	0.22	0.85	0.07
XI-2	5,417,000	5,965,000	0.97	0.64	0.96	0.75	0.82	0.99	0.09	0.99	0.56	0.70	0.10
XI-3	8,951,000	8,986,000	0.54	0.66	0.37	0.52	0.50	0.93	0.72	0.64	0.65	0.42	0.10
XI-4	9,033,000	9,056,000	0.99	0.63	0.85	0.43	0.34	0.31	0.08	0.66	0.96	0.19	0.14
XI-5	9,745,000	9,778,000	0.99	0.98	1.00	0.00	0.00	0.99	0.33	0.84	0.00	0.89	0.08
XII-1	2,165,000	2,217,000	1.00	0.11	0.97	0.06	0.07	0.93	0.70	0.25	0.68	0.50	0.09
XII-2	14,111,000	14,234,000	0.29	0.31	0.66	0.38	0.38	0.77	0.62	0.70	0.47	0.64	0.11
XII-3	14,299,000	14,391,000	0.96	0.95	0.96	0.97	0.96	0.98	0.90	0.93	0.96	0.69	0.13
XII-4	14,425,000	14,492,000	0.20	0.40	0.16	0.07	0.55	0.97	0.10	0.11	0.25	0.81	0.11
XII-5	16,490,000	16,547,000	1.00	1.00	1.00	0.92	1.00	1.00	1.00	0.99	1.00	1.00	0.15
XIII-1	8,442,000	8,464,000	0.99	0.97	0.99	0.81	0.99	0.64	0.90	0.95	0.99	0.59	0.11
XIV-1	11,338,000	11,371,000	1.00	0.88	0.90	0.75	0.85	1.00	0.03	0.97	0.67	0.92	0.15
XV-1	7,185,000	7,210,000	0.99	0.49	1.00	0.78	0.46	0.97	0.12	0.97	0.79	0.72	0.15
XVI-1	4,031,000	4,117,000	0.30	0.42	0.21	0.26	0.28	0.88	0.14	0.15	0.20	0.76	0.10
XVI-2	13,825,000	13,848,000	0.29	0.11	0.41	0.09	0.14	0.99	0.10	0.15	0.20	0.34	0.06
XVIII-1	834,000	914,000	0.62	0.77	0.71	0.81	0.61	0.56	0.61	0.76	0.52	0.92	0.13
XIX-1	2,440,000	2,601,000	0.98	0.97	0.99	0.98	0.98	0.97	0.98	0.96	0.98	0.97	0.13
XIX-2	14,750,000	14,816,000	0.84	0.82	0.94	0.90	0.84	0.80	0.55	0.74	0.76	0.82	0.13
XX-1	171,000	287,000	0.46	0.22	0.98	0.00	0.77	0.06	0.05	0.19	0.94	0.96	0.01
XX-2	3,590,000	3,623,000	1.00	0.93	0.97	0.57	0.98	0.94	0.92	0.67	0.98	0.88	0.15
XX-3	8,595,000	8,661,000	1.00	0.80	0.99	0.97	0.79	0.33	0.55	0.85	1.00	0.82	0.13
XX-4	8,865,000	9,014,000	0.73	0.77	0.98	0.64	0.97	0.47	0.97	0.95	0.97	0.86	0.15
XX-5	10,906,000	10,950,000	0.80	0.81	0.54	0.63	0.88	0.64	0.78	0.55	0.72	0.69	0.09
XX-6	11,716,000	11,742,000	1.00	0.84	0.69	1.00	0.52	0.89	0.63	0.97	0.78	0.64	0.18
XXI-1	5,728,000	7,495,000	0.35	0.41	0.46	0.81	0.59	0.80	0.67	0.37	0.69	0.29	0.04
XXI-2	7,811,000	8,082,000	0.49	0.59	0.89	0.79	0.82	0.57	0.61	0.64	0.79	0.85	0.11

Table S5. List of DIs with corresponding freshwater allele frequencies in the populations studied.

DI	Freshwater allele frequency	Rigidity	Marker density	Pervasive ness	Length	$4N_er$ (Feulner et al. 2015)	Core length	Periphery length
I-1	0.88	0.98	0.007	0.3	36,000	3.08	35,000	1,000
I-2	0.78	0.65	0.002	0.4	166,000	16.20	22,000	144,000
I-3	0.76	0.91	0.002	0.6	44,000	3.46	0	44,000
I-4	0.99	0.99	0.014	1	515,000	3.26	483,000	32,000
I-5	0.82		0.003	0.2	22,000	1.52	22,000	0
II-1	0.80	0.95	0.002	1	146,000	4.44	32,000	114,000
II-2	0.77	0.88	0.004	1	82,000	1.39	9,000	73,000
II-3	0.67	1.00	0.003	0.7	21,000	6.57	21,000	0
III-1	0.77	0.36	0.003	0.2	38,000	5.97	19,000	19,000
III-2	0.73	0.17	0.002	0.3	69,000	40.99	0	69,000
III-3	0.78	0.89	0.003	0.6	28,000		16,000	12,000
III-4	0.78	0.84	0.003	0.5	29,000	3.99	16,000	13,000
IV-1	0.82	0.98	0.003	1	31,000	1.33	21,000	10,000
IV-2	0.96	0.99	0.010	1	98,000	2.31	94,000	4,000
IV-3	0.95	0.95	0.006	1	122,000	3.36	39,000	83,000
IV-4	0.77	1.00	0.002	1	37,000	4.14	16,000	21,000
IV-5	0.98	0.99	0.008	1	120,000	0.87	115,000	5,000
IV-6	0.77		0.004	0.2	23,000	2.67	23,000	0
IV-7	0.83	0.99	0.008	1	82,000	4.51	40,000	42,000
IV-8	0.75	0.95	0.005	0.7	193,000	5.90	186,000	7,000
IV-9	0.80	0.94	0.002	0.6	108,000	4.77	0	108,000
V-1	0.87	0.95	0.007	1	55,000	11.66	18,000	37,000
VII-1	0.79	0.70	0.003	0.4	59,000		16,000	43,000
VII-2	0.73		0.002	0.3	22,000	63.52	20,000	2,000
VII-3	0.98		0.003	0.1	36,000	16.21		
VII-4	0.86	1.00	0.002	1	30,000	2.57	23,000	7,000
VII-5	0.71	0.95	0.006	0.7	76,000	2.31	18,000	58,000
VII-6	0.85	1.00	0.009	0.6	42,000	3.90	40,000	2,000

VIII-1	0.82	0.98	0.006	0.5	35,000	3.37	34,000	1,000
VIII-2	0.66	0.99	0.003	0.8	81,000	3.74	31,000	50,000
IX-1	0.73	0.87	0.005	0.9	129,000	3.81	0	129,000
IX-2	0.70	0.73	0.005	0.9	47,000	5.18	8,000	39,000
IX-3	0.95	1.00	0.003	0.9	86,000	3.27	17,000	69,000
IX-4	0.82	0.93	0.002	0.8	115,000	9.07	18,000	97,000
IX-5	0.68	0.99	0.002	0.5	39,000	0.93	29,000	10,000
IX-6	0.86	0.98	0.003	1	124,000	7.84	30,000	94,000
IX-7	0.66	0.75	0.002	0.9	122,000	5.02	0	122,000
X-1	0.67		0.002	0.3	60,000	9.03	21,000	39,000
X-2	0.62	0.06	0.001	0.3	106,000	11.02	0	106,000
XI-1	0.83	0.68	0.002	0.3	222,000	9.73	112,000	110,000
XI-2	0.82	0.88	0.010	0.9	548,000	9.78	434,000	114,000
XI-3	0.67		0.002	0.7	35,000	8.95	17,000	18,000
XI-4	0.82	1.00	0.004	0.5	23,000	5.52	22,000	1,000
XI-5	0.95	0.73	0.003	0.6	33,000		9,000	24,000
XII-1	0.80	0.99	0.006	0.6	52,000	2.04	22,000	30,000
XII-2	0.68	0.72	0.001	0.5	123,000	3.40	24,000	99,000
XII-3	0.93	0.96	0.004	1	92,000	3.71	31,000	61,000
XII-4	0.78	0.33	0.003	0.3	67,000	3.38	38,000	29,000
XII-5	0.99	0.99	0.002	1	57,000	2.71	34,000	23,000
XIII-1	0.88	1.00	0.003	1	22,000	0.34	21,000	1,000
XIV-1	0.88	0.99	0.006	0.9	33,000	5.33	29,000	4,000
XV-1	0.89	1.00	0.004	0.7	25,000	2.15	18,000	7,000
XVI-1	0.82	0.94	0.002	0.2	86,000	5.51	22,000	64,000
XVI-2	0.99		0.003	0.1	23,000			
XVIII-1	0.69	0.81	0.003	1	80,000	5.62	0	80,000
XIX-1	0.98	0.99	0.008	1	161,000		153,000	8,000
XIX-2	0.80	0.97	0.003	1	66,000		60,000	6,000
XX-1	0.91	1.00	0.008	0.4	116,000	2.46	113,000	3,000
XX-2	0.88	0.98	0.004	1	33,000	5.83	26,000	7,000
XX-3	0.86	1.00	0.004	0.9	66,000	7.26	25,000	41,000

XX-4	0.87	0.98	0.002	0.9	149,000	3.78	34,000	115,000
XX-5	0.70	0.77	0.003	1	44,000	7.64	18,000	26,000
XX-6	0.80	1.00	0.002	1	26,000	3.83	22,000	4,000
XXI-1	0.71	0.78	0.003	0.5	1,767,000	1.76	1,709,000	58,000
XXI-2	0.73	0.80	0.002	0.9	271,000	7.50	77,000	194,000

Table S6. Properties of the 65 identified DIs. Freshwater allele frequency is calculated as a mean frequency among populations in which DI has responded. Pervasiveness corresponds to the fraction of populations in which the DI has reached freshwater allele frequency > 0.5. Core region length is not defined for DIs VII-3 and XVI-2, which have responded in only one population.

	LN	CAN	KUM	LOB	MAS	SON	MAS3	OG	BG	VOR
Distance <1Mb	0.22	0.26	0.27	0.37	0.37	0.31	0.32	0.26	0.26	0.32
Distance > 1Mb	0.33	0.27	0.27	0.27	0.29	0.33	0.37	0.28	0.23	0.26
P-value	0.04	0.70	0.58	0.98	0.98	0.45	0.17	0.52	0.61	0.79

Table S9. Differences in allele frequency for pairs of remote DIs (located on distance > 1Mb, same chromosome) are usually no greater than allele frequency differences for pairs of nearby DIs (located <1Mb). First and second rows, averages for absolute values of allele frequency differences. P-values obtained by applying one-sided Wilcoxon test.
